# Association between Acquired Uniparental Disomy and Homozygous Mutations and HER2/ER/PR Status in Breast Cancer

**DOI:** 10.1371/journal.pone.0015094

**Published:** 2010-11-30

**Authors:** Musaffe Tuna, Marcel Smid, Dakai Zhu, John W. M. Martens, Christopher I. Amos

**Affiliations:** 1 Department of Epidemiology, The University of Texas M. D. Anderson Cancer Center, Houston, Texas, United States of America; 2 Department of Medical Oncology, Erasmus Medical Center Josephine Nefkens Institute and Cancer Genomics Center, Rotterdam, The Netherlands; Health Canada, Canada

## Abstract

**Background:**

Genetic alterations in cellular signaling networks are a hallmark of cancer, however, effective methods to discover them are lacking. A novel form of abnormality called acquired uniparental disomy (aUPD) was recently found to pinpoint the region of mutated genes in various cancers, thereby identifying the region for next-generation sequencing.

**Methods/Principal Findings:**

We retrieved large genomic data sets from the Gene Expression Omnibus database to perform genome-wide analysis of aUPD in breast tumor samples and cell lines using approaches that can reliably detect aUPD. aUPD was identified in 52.29% of the tumor samples. The most frequent aUPD regions were located at chromosomes 2q, 3p, 5q, 9p, 9q, 10q, 11q, 13q, 14q and 17q. We evaluated the data for any correlation between the most frequent aUPD regions and HER2/*neu*, ER, and PR status, and found a statistically significant correlation between the recurrent regions of aUPD and triple negative (TN) breast cancers. aUPD at chromosome 17q (*VEZF1*, *WNT3*), 3p (*SUMF1*, *GRM7*), 9p (*MTAP*, *NFIB*) and 11q (*CASP1*, *CASP4*, *CASP5*) are predictors for TN. The frequency of aUPD was found to be significantly higher in TN breast cancer cases compared to HER2/*neu*-positive and/or ER or PR-positive cases. Furthermore, using previously published mutation data, we found *TP53* homozygously mutated in cell lines having aUPD in that locus.

**Conclusions/Significance:**

We conclude that aUPD is a common and non-random molecular feature of breast cancer that is most prominent in triple negative cases. As aUPD regions are different among the main pathological subtypes, specific aUPD regions may aid the sub-classification of breast cancer. In addition, we provide statistical support using *TP53* as an example that identifying aUPD regions can be an effective approach in finding aberrant genes. We thus conclude that a genome-wide scale analysis of aUPD regions for homozygous sequence alterations can provide valuable insights into breast tumorigenesis.

## Introduction

Genetic mutations are the hallmark of cancer. High-density genome-wide analyses of biological samples using conventional high-throughput comparative genomic DNA microarrays discern recurrent DNA copy-number (CN) alterations *i.e.,* gains or losses from acquired uniparental disomy (aUPD) regions in the cancer genome. The availability of genome-wide single-nucleotide polymorphism (SNP) genotype-array technology and suitable analytical tools has revealed the presence of aUPD, which has now been recognized in various cancers [1]–[12], and it can pinpoint regions that contain homozygously mutated, methylated, or imprinted genes.

Understanding the molecular pathogenesis of cancer requires detailed cataloguing of all genetic and epigenetic lesions—not just identification of CN changes, but also detection of aUPD, DNA sequence, and methylation changes—in cancer cells. Because of the previous lack of high-throughput technology and analytical tools, to date very few reports have been published in breast cancer about either genome-wide aUPD analysis [5] or aUPD for specific genes, such as *RB1* and *TP53* [1]. We now know that aUPD can occur either on the entire chromosome or segmentally: a loss of one chromosome followed by duplication of the remaining chromosome leads to aUPD on the entire chromosome, whereas somatic recombination leads to segmental UPD [13]–[15]. Both mechanisms lead to the transmission to the daughter cell of a homozygous mutation from a heterozygous parental cell. The regions having aUPD are evident as large CNN stretches of somatically acquired homozygosity without any change in DNA content.

The distribution of aUPD regions appears non-random, and homozygous gene mutations have been discovered in aUPD regions in various cancers [6], [10], [11], [16], [17]. For example, associations have been found between aUPD and homozygous mutations: in *c-CBL* in acute myeloid leukemia and atypical chronic myeloid leukemia [17], [18]; in *JAK2* and myeloproliferative disorders [2], [19]; in *NF-1* and juvenile myelomonocytic leukemia [20]; in *A20* and B-cell lymphoma [10], and in *TET* and myelodysplastic syndrome [3], [11], in *MPL* and refractory anemia with ringed sideroblasts and thrombocytosis [6]; in *c-KIT, WT1*, and *PTPN11* and acute lymphoblastic leukemia [21]–[23]; and in *CEBPA* or *AML1/RUNX1* and acute myeloid leukemia [22], [24], [25]. aUPD is also clinically relevant, as shown by the association between clinical outcome and aUPD in follicular lymphoma [9] and glioblastoma multiforme [26].

aUPD may result in two copies of an abnormal allele, which may give a growth advantage to the cell. Some of these abnormalities or mutations may affect mRNA- and protein-expression levels. Homozygously mutated genes in aUPD regions that function in the initiation and progression of cancer may be associated with tumor type or subtype [8], [27], risk of disease transformation [9], patient's survival time [9], [28]. Inactivation of genes through different mechanisms may lead to or occur in different subtypes of disease. For example, in uveal melanomas, monosomy at chromosome 3 results in pigmented tumors, whereas aUPD at chromosome 3 results in unpigmented tumors [8]. Thus the dysfunction of cellular processes caused by deletion of a gene may affect a different cellular pathway than that affected by aUPD in the same gene.

As a result of all these findings, we hypothesized that aUPD is also a common feature found in breast cancer. Since genome-wide aUPD analysis by using high-resolution SNP arrays can pinpoint regions that carry homozygously mutated genes for next-generation gene sequencing, we hypothesized that identifying UPD regions can identify known and possibly novel mutated genes in breast cancer.

Breast cancers are routinely assessed for the expression of ER, PR and overexpression or amplification of the HER2/*neu*. Patients with HER2/*neu*-positive tumors (30%) respond to treatment with the anti-HER2 monoclonal antibody transtuzumab [29]. Patients with ER- or PR- positive tumors are candidates for hormonal therapy, including selective ER modulators such as tamoxifen for premenopausal women or aromatase inhibitors for postmenopausal women [30], [31]. Patients with triple negative cancers (those negative for ER, PR and HER2) currently have no available targeted therapy and have relatively poor prognosis [32], [33].

As the biology resulting in breast cancer pathological subtypes is different, we further hypothesized that specific aUPD regions might correlate with estrogen receptor (ER), progesterone receptor (PR), and/or HER2/*neu* status.

Our purpose in conducting this analysis was to identify aUPD regions in breast cancer samples. Such regions might be candidate regions for second-generation sequencing to identify novel mutated genes in breast cancer. This study is the first, to our knowledge, to describe high-resolution genome-wide UPD analysis of a large dataset and its integration with sequence alterations of *TP53* in breast tumor samples.

The findings presented here provide strong evidence that mitotic recombination is a common molecular mechanism that results in an aUPD feature that occurs non-randomly in specific chromosomal locations, and that correlates with ER, PR and HER2/*neu* status of breast cancer and with homozygous mutation of specific genes.

## Materials and Methods

We conducted analysis to identify genome-wide aUPD regions using data from 700 breast tumor samples and cell lines. The analyses were conducted using AsCNAR/CNAGv3 software (http://genome.umin.jp) [34]. The raw data (CEL files) of the Affymetrix GeneChip DNA-mapping microarrays from six sets of breast cancer samples; GSE3743 [5], GSE7545 [35], GSE10099 [36], GSE16619 [37], GSE19399 [38] and GSE13696) [39] were retrieved from the Gene Expression Omnibus (GEO) database (http://www.ncbi.nih.nlm.gov/geo). The analysis was done by using non-self controls with sex-matched reference samples from HapMap data and from previously published, publicly available datasets; GSE14656 [40], GSE14860 [41], GSE10922 [42], GSE11417 [43], GSE10092 [44], GSE9611 [45], GSE9845 [46], GSE7946 [47], GSE15526 [48], GSE12702 [49] and GSE8333 [50]. The presence of aUPD regions was predicted by using a Hidden-Markov Model with default parameters as previously described Nannya [34], [51]. In the aUPD analyses both the genotype information and the intensity were used [34], [51]. The aUPD-score was calculated by counting the total number of aUPD regions in all chromosomes in each sample. The May 2006 human genome browser (NCBI Build 36/hg18); http://genome.ucsc.edu) was used for identifying gene localization and function. Gene mutation data for this analysis were retrieved from the Catalogue of Somatic Mutations in Cancer (COSMIC) database (http://www.sanger.ac.uk/genetics/CPG/cosmic) and from the study reports of Hu *et al.*, Hollestelle *et al.*, Sjoblom *et al.*, Wood *et al.,* and Stephens *et al.* [39], [52], [53], [54], [55].

We first identified genome-wide aUPD in all breast tumor samples (n = 656) and cell lines (n = 44), then we performed correlation analysis between aUPD regions and ER, PR and HER2/*neu* status of those samples that had such data available (n = 467) of which 111 of them are TN. Pathologic characteristics of tumors are summarized in **Table S1**.

We used Fisher's exact test calculated by STATAv10 (StataCorp, CollegeStation, TX, USA) and Spearman correlation analyses by SAS v9.2 (SAS, NC, USA) to evaluate correlations between aUPD regions and ER, PR and HER2/*neu* status, grade, lobular or ductal, invasive or infiltrating pathology. The Wilcoxon Mann-Whitney test was used for testing the association of aUPD-scores with ER, PR and HER2/*neu* status. Stepwise logistic regression analyses was performed using SAS v9.2 (SAS, NC, USA) for prediction of pathology outcomes such as TN, ER, PR, and HER2/*neu* status, and Spearman correlation analyses were used for correlation between aUPD at chromosome 17p and *TP53* mutation status.

## Results

### Recurrent aUPD Regions in Breast Tumors

**Figure 1:**
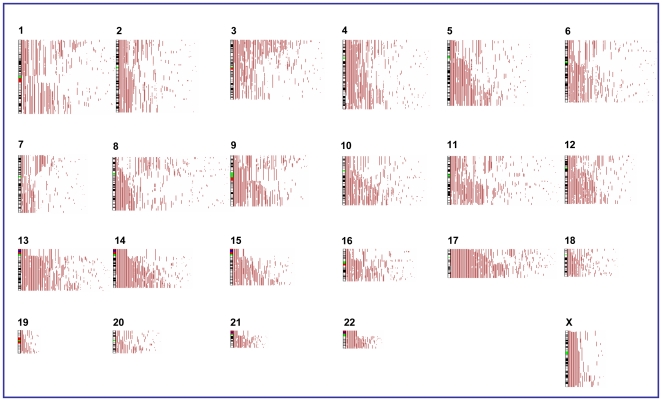
Distribution of aUPD in breast cancer samples.

To study the distribution of aUPD, our analysis included data on 656 tumor samples and 44 cell lines. aUPD was identified in 52.3% of tumor samples (343 of the 656) (**Figure 1**) and 100% of cell lines (**Figure S1**). Segmental aUPD was found more frequently (94.5% in tumors and 90.3% in cell lines) than whole-chromosome aUPD (5.5% in tumors and 9.7% in cell lines), suggesting that somatic recombination is a more frequent event in breast tumorigenesis than is loss of a chromosome followed by duplication of the remaining chromosome. The aUPD-score ranged from 0–64 in tumors (with median 2 and mean 4.85), and 1–47 in cell lines (with median 14 and mean 15.1). The most frequently aUPD region was observed at chromosome 17q (32.9%), while the least commonly affected chromosome was at chromosome 19. Further, recurrent aUPD was seen at chromosomes 13q (19.5%), 3p (18.1%), 2q (16.0%), 5q (15.2%), 11q (14.0%), 14q (12.8%) and 10q (12.5%), 9p (9.9%) and 9q (7.6%) in those 343 tumor samples (**Figure 1**), while 17q and 5q (52.3%), 14q (50.0%), 3p and 9q (45.5%), 10q (43.2%), 11q (40.9%), 13q (34.1%), 2q (31.8%) and 9p (22.7%) in cell lines (**Figure S1**). These data indicate that multiple genes may be targeted by aUPD and such regions may also be affected by other molecular events such as homozygous mutations, promoter methylation, histone modification or imprinting.

### Correlation between the regions of aUPD and ER, PR, HER2/*neu* and Status and pathological features of Breast Tumors

We next examined the data to identify any correlation between the recurrent aUPD regions of diverse clinical parameters (including ER, PR and HER2/*neu* status, grade, lobular or ductal subtype and invasive or infiltrating cancer), and characterized by a distinct aUPD profile and whether specific aUPD regions in each group of tumors harbor narrow regions of aUPD that indicate regions harboring homozygous mutation that may be used as a marker or a therapeutic target in each group of breast cancer. We found correlation with ER, PR and Her2/*neu* status (**Table 1**), grade and invasive type of cancer, but we could not find any correlation between aUPD regions and lobular or ductal type and infiltrating breast cancer (**Table S2**).

**Table 1:** Correlation between ER, PR and HER2/*neu* status and aUPD regions.

aUPD at chromosome	Correlation value for ER-	ER-	Correlation value for PR-	PR-	Correlation value for HER2-	HER2-	Correlation value for TN	TN
17q	0.34409	<0.0001	0.21243	<0.0001	0.20194	<0.0001	0.40810	<0.0001
13q	0.26440	<0.0001	0.17760	0.0001	0.17139	0.0003	0.32025	<0.0001
3p	0.23957	<0.0001	0.16660	0.0003	0.10408	0.0283	0.31768	<0.0001
11q	0.21998	<0.0001	0.21998	<0.0001	0.20037	0.0072	0.28533	<0.0001
2q	0.15471	0.0008	0.18000	<0.0001	0.07681	0.1060	0.26393	<0.0001
5q	0.11715	0.0114	0.08422	0.0696	0.11276	0.0175	0.15652	0.0010
14q	0.13214	0.0043	0.10348	0.0257	0.15518	0.0010	0.21263	<0.0001
9q	0.11761	0.0111	0.12040	0.0094	0.13369	0.0048	0.20023	<0.0001
9p	0.15705	0.0007	0.13386	0.0038	0.07558	0.1118	0.21667	<0.0001
10q	0.16653	0.0003	0.10744	0.0205	0.09748	0.0401	0.21263	<0.0001
Total aUPD-score	0.30503	<0.0001	0.27016	<0.0001	0.12360	0.0091	0.35755	<0.0001

*Correlation analyses were performed by using Spearmen correlation test.

Data on ER, PR, and HER2/*neu* status were available for 468 cases. We tested those data for any correlation with the presence of the most recurrent aUPD regions at chromosome 2q, 3p, 5q, 9p, 9q, 10q, 11q, 13q, 14q, 17q and total aUPD scores. We also assessed whether there was any correlation between the presence of recurrent aUPD regions and triple-negative tumors (n = 111) compared to tumors expressing at least one of the three receptors (ER, PR or HER2/*neu*) (n = 356),

aUPD at chromosome 17q and 13q revealed highly statistically significant association with ER- negative, PR- negative, HER2/*neu*-negative and TN cases (*P*<0.001) (**Table 1**), while all recurrent aUPD regions and total aUPD-score were highly statistically significant correlation with TN cases (*P*<0.001) (**Table 1**). Other recurrent aUPD regions had less significant association with ER-negative, PR-negative, and HER2/*neu*-negative cases (**Table 1** **,** **Figure 1** **–** **3**).

**Figure 2:**
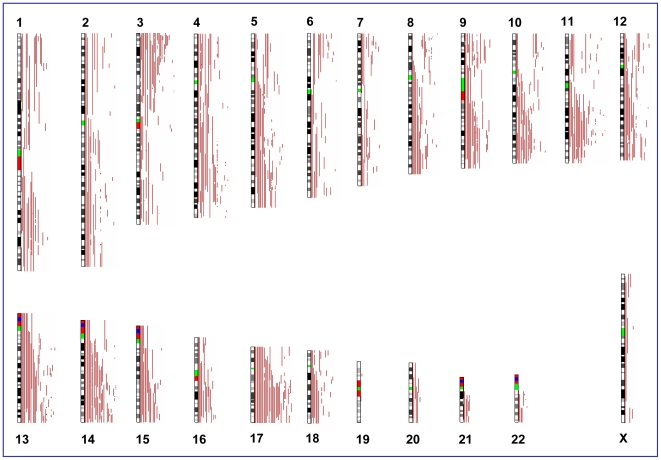
Distribution of aUPD in triple negative breast cancer samples.

**Figure 3:**
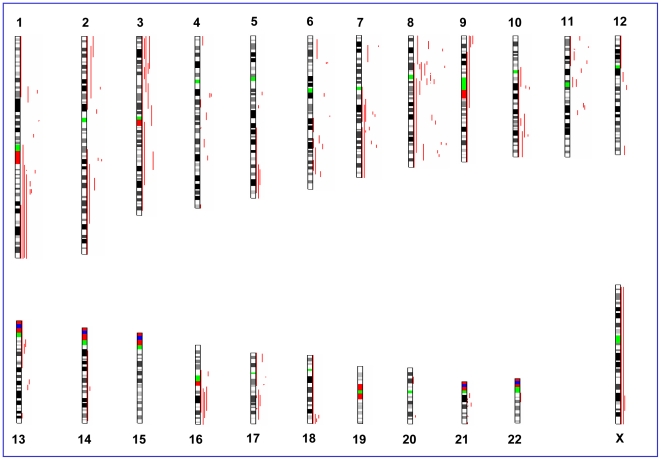
Distribution of aUPD in HER2/neu-positive breast cancer samples.

In addition, at a significance level of p<0.001 we observed differences in aUPD scores between ER-negative, PR-negative and HER2/*neu*-negative samples compared to their respective positive counterparts (**Table 1**, **Table S3**). Higher aUPD-scores were also found in TN-negatives compared to receptor positive counterparts (p<0.001) (**Table 1**). Similar results to those found in the clinical breast cancer specimens were seen in the breast cancer cell lines (**Figure S2, Figure S3**).

We also found that grade 3 pathology associated with aUPD at chromosome 17q, 13q and total aUPD-score (p<0.01), and invasive breast cancer with aUPD at chromosome 5q (p<0.05) (**Table S2**).

### aUPD predicts tumor characteristics and subtypes

We next assessed whether aUPD regions predicted tumor pathological characteristics and subtypes. Stepwise logistic regression analyses was performed to identify which aUPD regions independently predict tumor characteristics and subtypes, and we found that aUPD at chromosome 17q (*P*<0.001), 3p, 9p and 11q (p<0.05) were significant independent predictors for TN cases (**Table 2**), while 17q (*P*<0.001), 13q (*P*<0.01) and 3p (*P*<0.05) were predictors of ER-negative cases, 11q (*P*<0.05) are predictors for PR-negative cases, and 17q (*P*<0.001) and13q (*P*<0.01) are predictors for HER2/*neu*-negative cases (**Table S4**) These findings indicate TN breast tumors have different patterns of aUPD than those that are receptor positive suggesting that aUPD may be used for sub-classification of breast cancer.

**Table 2:** aUPD regions as predictors for triple negative breast cancer.

Tumor type	aUPD at chromosome	*P*
TN	17q	1.21E-06
TN	3p	0.0141
TN	9p	0.0418
TN	11q	0.0134

TN: triple negative; Multiple stepwise logistic regression was used to identify independent predictors of TN status.

In order to define candidate regions that may harbor specific genes relevant to breast cancer development, we identified the smallest aUPD regions at chromosome 3p21.31 (*TUSC4*, *SEMA3F*, *SEMA3B*, *RASSF1*, *MAPKAPK3*, *HYAL1*, *HYAL2*, *FUS*), 3p26.2-p26.1 (*LRRN1*, *SETMAR*, *SUMF1*), 3p26.1 (*GRM7*), 11q22.3 (*CASP*, *CASP4, CASP5*, *CASP12*, *COP1*, *CARD16*, *CARD18*, *GRIA4*) (**Table 3**) in TN breast cancer samples. Segmental aUPD occurs with mitotic recombination error, which leads to homozygous region and usually no copy number changes, however, in some cases one of these two alleles loss and remaining allele can be amplified which leads to focal amplification (**Figure S4**) or deleted allele can be reduplicated which causes focal homozygous deletion (**Figure S5**). Therefore, aUDP regions that carry focal amplifications may harbor homozygous oncogenic mutation(s). The focal amplification identified in aUPD regions were at chromosome 3p22.2 (*ITGA9*, *DLEC1*), 10q26.12-q26.13 (*FGFR2* and *K-sam*), 17q21.31-q21.32 (*WNT3*), 17q22 (*VEZF1*, *SFRS1*) (**Table 3** **, Figure S4**). In addition, homozygous deletions within aUPD regions, which indicates these regions may harbor homozygous mutated tumor suppressor genes, were found in chromosome 3p14.2 (*FHIT*), 9p23-p22.3 (*NFIB*), 9p21.3 (*CDKN2A*, *CDKN2B*, *miR-31*) (**Table 3**, **Figure S5**) in triple negative cases.

**Table 3:** The smallest recurrent aUPD regions in TN tumor samples.

Chromosomal Band	Start-End Positions	Length (bp)	Possible Candidate Genes	Possible Candidate miRNA
3p26.2-p26.1	3,500,000–4,500,000	1,000,001	*SUMF1*	
3p26.1	5,750,000–7,500,000	1,750,001	*GRM7*	
3p22.2#	37,479,869–38,325,230	845,362	*ITGA9*, *DLEC1*	*miR-26a-1*
3p22.1	42,071,245–43,557,137	1,485,893	*TRAK1*, *CCK*, *LYZL4*, *VIPR1*, *SEC22C*, *SS18L2*, *NKTR*, *ZBTB47*, *KBTBD5*, *HHATL*, *CCDC13*, *HIGD1A*, *CCBP2*, *CYP8B1*, *ZNF662*, *C3ORF39*, *SNRK*, *ANO10*	
3p21.31	49,926,113–51,159,065	1,232,953	*TUSC4*, *SEMA3F*, *SEMA3B*, *RASSF1*, *MAPKAPK3*, *HYAL1*, *HYAL2*, *FUS1*	*miR-566*
3p14.2*	59,585,338–60,009,495	424,158	*FHIT*	
9p23-p22.3*	13,531,695–14,417,560	885,866	*NFIB*	
9p21.3*	21,948,524–23,513,491	1,564,968	*MTAP*, *CDKN2A*, *CDKN2B*, *DMRTA1*	
9p21.3-p21.2*	21,014,103–25,983,972	4,969,870	*CDKN2A*, *CDKN2B*, *TUSC1*	*miR-31*
10q26.12-q26.13#	122,580,540–123,648,119	1,067,580	*FGFR2*, *K-sam*	
11q22.3	103,938,480–105,262,960	1,324,481	*CASP1*, *CASP4*, *CASP5,CASP12*, *CARD16*, *COP1*, *GRIA4*	
17q21.31-q21.32#	41,546,868–42,234,514	687,647	*WNT3*	
17q22#	53,292,386–53,489,452	197,067	*VEZF1*, *SFRS1*	

*Homozygous deletion,

# Amplification.

### Association between aUPD Regions and Homozygously Mutated Genes in Breast Tumors

To date, mutations have been found in a number of genes in breast cancer. However, the most important problems interpreting mutations is the presence of numerous mutations that have no direct role in cancer; these may be called ‘passenger mutations’. The other group of mutated genes, which affect protein function and involve tumor initiation and/or progression, may be called ‘drivers.’ Distinguishing the driver genes from the passengers is challenging, but the integration of aUPD analysis with mutation and functional data can overcome this problem.

We decided to evaluate the known relevance of *TP53* as the most frequently mutated gene [39], [56] with our aUPD data of breast cancer cell lines to test whether aUPD correlates with homozygous mutation in a known gene that localized in that region. We found a strong correlation (r = 0.48441, *P* = 0.0012) between homozygous mutation of *TP53* and aUPD at chromosome 17p (**Figure S6**) region covering the *TP53* locus (22/26). In contrast, we did not see a significant association between homozygous mutation of *PIK3CA* (1/7), *CDKN2A* (2/6), *PTEN* (5/12), *RB1* (2/9) and *CDH1* (2/10) genes and their localized regions, perhaps because, some of these genes (*CDH1*, *CDKN2A*, *PTEN*) undergo suppression of function by hypermethylation (**Table S5**). Nevertheless, the presence of some aUPD for each of these cell lines suggests that aUPD may provide an important tool for discovering genes important in tumorigenesis.

Then, we integrated previously identified homozygous-mutation data in tumor samples [52], [53], [54], [56], [57] into our analysis, and found that homozygously mutated genes (**Table S6**) also localize in the aUPD regions (**Figure S7**). The most common mutations found in breast cancer are reported regardless of homozygosity or heterozygosity at *TP53* (44–47%), *PIK3CA* (25–26%), *CDH1* (21–22%), *PTEN* (5–6%), and *CDKN2A* (4–5%) [56]. Previously reported homozygously mutated genes are summarized in **Table S6**; these genes also mapped in the aUPD regions we identified. Those homozygously mutated genes function in apoptosis (*e.g., ATR*, *CDKN2A*), the cell cycle or cell proliferation (*e.g., BRCA1*, *TP53*, *CDKN2A*, *PTEN*, *TAF1*), and cell adhesion (*e.g.*, *ARHGEF4*, *CDH1*, *ICAM5*, *PCDHB15*). Some of those genes are already well-known as tumor-suppressor genes (*e.g., APC*, *BRCA1*, *CDKN2A*, *PTCH1*, *PTEN*, *SMAD4*, *TP53*), and oncogenes (*e.g., FGFR1*, *MET*, *PIK3CA*) (**Table S6**) and genes that tyrosine kinases can use as a therapeutic target (*e.g., PIK3CA*, *AKT*, *FGFR1*) and all mapped at aUPD regions we observed.

If indeed aUDP pinpoints these aberrant genes, the latter findings with these integrated data indicate that more than one cell-signaling pathway is being interrupted in breast cancer.

## Discussion

In this study, using a large representative cohort of patients with breast cancer (n = 656) and cell lines (n = 44), we have shown that aUPD is a common and non-random event in breast tumorigenesis. The frequency of aUPD is statistically significantly higher in TN breast cancer and in estrogen receptor negative than receptor positive tumors. We have characterized aUPD regions associated with the most reproducible breast cancer subtypes, defined by tumor ER, PR, and HER2/*neu* status. For clinical practice ER and PR status is generally established by immunohistochemistry (IHC), and HER2/*neu* status by IHC or fluorescence *in situ* hybridization. In addition, in the current study aUPD at 17q, 3p, 9p and 11q were found as predictors for TN cases, while aUPD at 17q, 13q and 3p were predictors of ER-negative disease. aUPD at 11q was predictive of PR-negative breast cancer, and aUPD at 17q and 13q marked HER2/*neu*-negative cases. Overall our findings indicate that each group has a different pattern and that specific aUPD regions clearly associated with ER, PR or HER2/neu status.

Until now sporadic breast tumors have shown mutations in different genes, with *TP53* being the most frequently mutated *(*44% in tumor and 73–76% in cell lines) [39], [55] particularly in *BRCA1* and sporadic basal-like carcinoma [58], [59]. It is also known that breast cancers in patients with *BRCA1* germ-line mutations are more often triple negative than positive for HER2/*neu*, PR or ER [60], and the majority of basal-like carcinomas lack ER, PR, and HER2/*neu* expression. In concordance with this finding, our result (**Figure 2**) provides strong evidence that in addition to mutation in *TP53* and *BRCA1*, other genes in aUPD region at chromosome 17q, 3p, 9p and 11q may be mutated or otherwise suppressed in triple-negative tumors. Thus it is possible that other than *TP53* and *BRCA1* mutated genes in these regions contribute functionally to the development of triple-negative breast cancers; future studies, however, are needed to support this finding.

One of the candidate genes for mutation is *VEZF1* at chromosome 17q, which is transcriptional regulatory zing finger protein 161. This gene regulates DNA methylation [61], [62] and is involved in both normal and abnormal cellular proliferation and differentiation. *WNT3* is in another aUPD region of chromosome 17q and is a member of WNT gene family. Gene expression studies suggest that this gene may play a key role in variety of human cancer including breast cancer through activation of the WNT-beta-catenin-TCF pathway, and the WNT pathway may be active in basal-like tumors relapsing to brain based on pathway analysis [63]. Another candidate gene is *miR-31* which is affected by the focal homozygous deleted region at chromosome 9p. Overexpression of *miR-31* inhibits breast cancer metastasis [64], suggesting that homozygous deletion of *miR-31* may play role in metastasis of breast cancer. A final candidate for mutation is *FGFR2* at chromosome 10q. *FGFR2* is a member of the fibroblast growth factor receptor family, showed heterozygous mutation in *FGFR2* in breast cancer [52], and recently showed that SNPs (rs2981582) in this gene associated with increased risk of breast cancer [65]. Allele-specific up-regulation of *FGFR2* was associated with increasing susceptibility to breast cancer [66]. We found aUPD at *FGFR2* region in chromosome 10q. Taken together, data indicates that *FGFR2* may be a good candidate for homozygous mutation or imprinting.

From all these data, we conclude that aUPD is a common and non-random molecular event in breast cancer. Identifying aUPD regions could be a very effective approach for discovering novel candidate genes for mutation screening. Our data also suggest that aUPD may be used for sub-classification of breast tumors. Finally, the integration of mutation data with aUPD data provides strong evidence that many more genes than previously thought to be aberrant in breast cancer and which await discovery and could include useful new therapeutic targets. aUPD may pinpoint regions with homozygous alterations and identifying those mutated genes will provide valuable insights.

## Supporting Information

Table S1 **Pathologic characteristics of breast tumors.**
(PDF)

Table S2 **Correlation between aUPD regions and grade, invasive, infiltrating, lobular and ductal type of breast cancer.**
(PDF)

Table S3 **aUPD regions as predictor of pathologic breast cancer characteristics and subtypes.**
(DOC)

Table S4 **Comparison of total aUPD-scores between ER^−^/PR^−^/HER2^−^ to their receptor positive counterparts.**
(DOC)

Table S5 **Mutation, deletion and aUPD of** ***TP53***, ***PIK3CA***, ***CDKN2A***, ***PTEN***, ***RB1*** **and** ***CDH1*** **genes.**
(DOC)

Table S6 **Previously reported homozygous mutated genes in breast cancer samples.**
(DOC)

Figure S1 **Distribution of aUPD regions in breast cancer cell lines (BrCaCL).**
(TIF)

Figure S2 **Distribution of aUPD regions in triple negative (BrCaCL).**
(TIF)

Figure S3 **Distribution of aUPD in HER2/neu-positive BrCaCL.**
(TIF)

Figure S4 **Representative smallest aUPD regions with focal amplification in TN samples.** The upper panel represents total copy number (log2 ratio), on the middle chromosome idiogram, and green bar in the middle represents heterozygous SNP calls in tumor. The lower panel represents allele-based changes. At the bottom panel genes (*FGFR2*, *ATE1* and *K-sam*) localized in the aUPD region at chromosome 10q26.12-q26.13.
(TIF)

Figure S5 Representative smallest aUPD regions with homozygous deletion in TN samples. The upper panel represents total copy number (log2 ratio), on the middle chromosome idiogram, and green bar in the middle represents heterozygous SNP calls in tumor. The lower panel represents allele-based changes.gene (*NFIB*) at chromosome 9p23-p22.3 from genome browser (UCSC).
(TIF)

Figure S6 **Representative figure for correlation of genomic and genetic data for three cell lines; MDAMB436, BT483 and CAL148.** The upper panel showed log2 ratio, middle panel shoed chromosome ideogram and SNP heterozygous bar (green), and lower panel showed allele-based changes. Dashed square represents aUPD region at chromosome 17p. The first cell line harbors homozygous mutation for TP53 and aUPD at the same region. The second cell line harbors homozygous mutation for TP53 and heterozygous deletion at the same region. The third cell lines harbor heterozygous mutation for TP53 and no copy number changes.
(TIF)

Figure S7 **Distribution of aUPD and localization of previously reported homozygously mutated genes in breast cancer samples.** Each line represents aUPD for each case. Each star represents previously reported homozygous mutated genes, which are also mapped in the aUPD regions in breast cancer.
(TIF)
