# Supplementary material for: Association between Acquired Uniparental Disomy and Homozygous Mutations and HER2/ER/PR Status in Breast Cancer
Source: PLoS One. 2010 Nov 30;5(11):e15094. doi: 10.1371/journal.pone.0015094 (PMC2994899; doi:10.1371/journal.pone.0015094)
Supplement: Table S1 — Pathologic characteristics of breast tumors. (PDF) [file pone.0015094.s001.pdf]

Table S1. Pathologic characteristics of breast tumors

Pathologic Characteristics	GSE10099 (n=313)	GSE16619 (n=161)	GSE7545 (n=51)	GSE3743 (n=41)	GSE19399 (n=90)
ER status					
Positive	199 (64%)	54 (33.5%)	6 (11.8%)	16 (39.0%)	na
Negative	114 (36%)	38 (23.6%)	11 (21.6%)	24 (58.5%)	na
Uknown	0	68 (42.2%)	34 (66.7%)	1 (2.4%)	na
PR status					
Positive	156 (49.8%)	51 (31.7%)	4 (7.8%)	14 (34.1%)	na
Negative	157 (50.2%)	41 (25.5%)	9 (17.6%)	26 (63.4%)	na
Unknown	0	69 (42.9%)	38 (74.5%)	1 (2.4%)	na
HER2/neu status					
Positive	79 (25.2%)	52 (32.3%)	10 (19.6%)	8 (19.5%)	na
Negative	234 (74.8%)	23 (14.3%)	5 (9.8%)	30 (73.2)	na
Unknown	0	86 (53.4%)	36 (70.6%)	3 (7.3%)	na
Grade					na
I	na	10 (6.2%)	na	0	na
II	na	47 (29.2%)	na	0	na
III	na	96 (59.6%)	na	41 (100%)	na
Unknown	na	8 (5.0%)	na	0	na
