# Supplementary material for: Association between Acquired Uniparental Disomy and Homozygous Mutations and HER2/ER/PR Status in Breast Cancer
Source: PLoS One. 2010 Nov 30;5(11):e15094. doi: 10.1371/journal.pone.0015094 (PMC2994899; doi:10.1371/journal.pone.0015094)
Supplement: Table S2 — Correlation between aUPD regions and grade, invasive, infiltrating, lobular and ductal type of breast cancer. (PDF) [file pone.0015094.s002.pdf]

Table S2. Correlation between aUPD regions and grade, invasive, infiltrating, lobular and ductal type of breast cancer.

Tumor type and Grade	aUPD at chromosome	*P*-value*
Invasive vs Infiltrating	17q	0.9355
Lobular vs Ductal	17q	0.3984
Invasion+ vs Invasion-	17q	0.7383
Grade 3 vs Grade 1&2	17q	**0.0021**
Invasive vs Infiltrating	13q	1.000
Lobular vs Ductal	13q	1.000
Invasion+ vs Invasion-	13q	1.000
Grade 3 vs Grade 1&2	13q	**0.0060**
Invasive vs Infiltrating	3p	0.7965
Lobular vs Ductal	3p	1.000
Invasion+ vs Invasion-	3p	1.000
Grade 3 vs Grade 1&2	3p	0.0555
Invasive vs Infiltrating	11q	0.1138
Lobular vs Ductal	11q	0.2484
Invasion+ vs Invasion-	11q	0.3332
Grade 3 vs Grade 1&2	11q	0.3849
Invasive vs Infiltrating	2q	0.7430
Lobular vs Ductal	2q	0.9633
Invasion+ vs Invasion-	2q	0.6818
Grade 3 vs Grade 1&2	2q	0.484
Invasive vs Infiltrating	5q	**0.0303**
Lobular vs Ductal	5q	0.9633
Invasion+ vs Invasion-	5q	0.3332
Grade 3 vs Grade 1&2	5q	0.1389
Invasive vs Infiltrating	14q	0.6818
Lobular vs Ductal	14q	0.6657
Invasion+ vs Invasion-	14q	0.2467
Grade 3 vs Grade 1&2	14q	0.2182
Invasive vs Infiltrating	9q	0.3388
Lobular vs Ductal	9q	0.4842
Invasion+ vs Invasion-	9q	0.6022
Grade 3 vs Grade 1&2	9q	0.0919
Invasive vs Infiltrating	9p	0.1702
Lobular vs Ductal	9p	0.8803
Invasion+ vs Invasion-	9p	0.5308
Grade 3 vs Grade 1&2	9p	0.3539
Invasive vs Infiltrating	10q	0.7979
Lobular vs Ductal	10q	0.2296
Invasion+ vs Invasion-	10q	0.9388
Grade 3 vs Grade 1&2	10q	0.3259

*Fisher's exact tests were used for evaluate correlations between aUPD regions and lobular or ductal, invasive or infiltrating, and Spearman correlation analyses used for evaluate correlations between aUPD regions and grade.
