# Supplementary material for: Association between Acquired Uniparental Disomy and Homozygous Mutations and HER2/ER/PR Status in Breast Cancer
Source: PLoS One. 2010 Nov 30;5(11):e15094. doi: 10.1371/journal.pone.0015094 (PMC2994899; doi:10.1371/journal.pone.0015094)
Supplement: Table S3 — aUPD regions as predictor of pathologic breast cancer characteristics and subtypes. (DOC) [file pone.0015094.s003.doc]

**Table S3**. aUPD regions as predictor of pathologic breast cancer characteristics and subtypes.

| **Tumor type** | **aUPD at chromosome** | ***P*** | **Predict** |
| --- | --- | --- | --- |
| ER+ vs ER- | 17q | 1.1E-13 | ER- |
| HER2+ vs HER2- | 17q | 2.09E-5 | HER2- |
| ER+ vs ER- | 13q | 0.0024 | ER- |
| HER2+ vs HER2- | 13q | 0.0239 | HER2- |
| ER+ vs ER^-^ | 3p | 0.0224 | ER- |
| PR+ vs PR- | 11q | 3.44E-0.6 | PR- |
| Total aUPD score | 11q | 0.0108 | PR- |

ER+; estrogen receptor positive, ER-; ER receptor negative, PR; progesterone receptor,

HER2; ERBB2. Stepwise logistic regression analyses were performed for prediction of

pathological subtype of breast cancer.
