# Supplementary material for: Association between Acquired Uniparental Disomy and Homozygous Mutations and HER2/ER/PR Status in Breast Cancer
Source: PLoS One. 2010 Nov 30;5(11):e15094. doi: 10.1371/journal.pone.0015094 (PMC2994899; doi:10.1371/journal.pone.0015094)
Supplement: Table S4 — Comparison of total aUPD-scores between ER−/PR−/HER2− to their receptor positive counterparts. (DOC) [file pone.0015094.s004.doc]

**Table S4.** Comparison of total aUPD-scores between ER^-^/PR^-^/HER2^-^ to their receptor positive counterparts

| **Number of sample** | **ER/PR/**  **HER2**  **status** | **Total**  **score** | **Mean**  **score** | **Median Score** | **Number of sample** | **ER/PR/**  **HER2**  **status** | **Total**  **score** | **Mean**  **score** | P | **Median Score** | P |
| --- | --- | --- | --- | --- | --- | --- | --- | --- | --- | --- | --- |
| 187 | ER- | 795 | 4.25 | 2 | 280 | ER+ | 421 | 1.51 | 8.71E-11 | 0 | 8.32E-08 |
| 248 | PR- | 897 | 3.62 | 1 | 218 | PR+ | 316 | 1.46 | 1.14E-08 | 0 | 3.48E-07 |
| 322 | HER- | 1027 | 3.19 | 1 | 123 | HER2+ | 147 | 1.20 | 0.014395 | 0 | 0.070189 |
| 111 | TN | 647 | 5.83 | 3 | 123 | HER2+ | 147 | 1.20 | 9.6E-10 | 0 | 2.78E-07 |
| 111 | TN | 647 | 5.83 | 3 | 51 | TP | 46 | 0.92 | 1.81E-07 | 0 | 6.98E-06 |
| 111 | TN | 647 | 5.83 | 3 | 184 | ER+&PR+ | 254 | 1.32 | 1.21E-14 | 0 | 8.03E-11 |
| 111 | TN | 647 | 5.83 | 3 | 344 | ER+/PR+/HER2+ | 552 | 1.60 | 1.04E-13 | 0 | 4.05E-09 |

ER-; estrogen negative, PR-; progesterone negative, HER2-; HER2/neu negative, ER+; estrogen positive, PR+, progesterone positive, HER2+; HER2/neu positive, TN; negative for all three receptors, TP; positive for all three receptor, comparison analyses of total aUPD-score between ER-/PR-/HER2 to their receptor positive counterparts were performed by Wilcoxon Mann-Whitney test.
