# Supplementary material for: Association between Acquired Uniparental Disomy and Homozygous Mutations and HER2/ER/PR Status in Breast Cancer
Source: PLoS One. 2010 Nov 30;5(11):e15094. doi: 10.1371/journal.pone.0015094 (PMC2994899; doi:10.1371/journal.pone.0015094)
Supplement: Table S5 — Mutation, deletion and aUPD of TP53, PIK3CA, CDKN2A, PTEN, RB1 and CDH1 genes. (DOC) [file pone.0015094.s005.doc]

**Table S5.** Mutation, deletion and aUPD of *TP53, PIK3CA, CDKN2A, PTEN, RB1* and *CDH1* genes.

|  |  | *TP53* | | *PIK3CA* | | *CDKN2A* | | *PTEN* | | *RB1* | | *CDH1* | |
| --- | --- | --- | --- | --- | --- | --- | --- | --- | --- | --- | --- | --- | --- |
| No | Cell lines | **Mut/met** | **aUPD/Del** | **Mut/Met** | **aUPD/Del** | **Mut/Met** | **aUPD/**  **Del** | **Mut/Met** | **aUPD/**  **Del** | **Mut/Met** | **aUPD/**  **Del** | **Mut/Met** | **aUPD/**  **Del** |
| 1 | HCC1806 | ND | aUPD |  |  | Hom | Het del |  |  |  | Het del | Met | Het del |
| 2 | AU565 | Hom | aUPD |  | Het del |  |  |  | Het del |  |  | Hom | Het del |
| 3 | BT474 | Hom | aUPD | Het |  |  | Het del |  | UPD |  | UPD |  |  |
| 4 | BT483 | Hom | Het Del | Hom |  |  |  |  | UPD |  |  |  |  |
| 5 | BT549 | Hom | aUPD |  | UPD |  |  | Hom | UPD | Hom | UPD |  | UPD |
| 6 | CAMA1 | Hom | Het Del |  |  |  |  | Het |  |  |  | Hom | UPD |
| 7 | DU4475 | NM |  |  |  |  |  |  | UPD | Hom | Hom del |  |  |
| 8 | HS578T | Hom | aUPD |  |  | Hom | Het del |  | Het del |  | Het del | Met | Het del |
| 9 | MCF7 | NM |  | Het |  | Hom | Het del |  |  |  | Het del |  |  |
| 10 | MDAMB175 | NM | Het  Del |  |  |  |  |  |  |  |  |  |  |
| 11 | MDAMB231 | Hom | aUPD |  |  | Hom | Hom del |  |  |  | Het del | Met | Het del |
| 12 | MDAMB361 | Hom | aUPD | Het |  | Hom | Het del |  |  |  | UPD |  |  |
| 13 | MDAMB415 | Hom | aUPD |  |  | Met |  | Hom | UPD |  | Het del |  |  |
| 14 | MDAMB436 | Hom | aUPD |  | Het del |  | Het del |  | Het del | Hom | Het del | Met | Het del |
| 15 | MDAMB453 | NM | Het Del | Het |  |  | Het del | Het |  |  | Het del | Hom | Het del |
| 16 | MDAMB468 | Hom | aUPD |  | UPD |  | Het del | Hom | UPD |  | Het del |  | UPD |
| 17 | SKBR3 | Hom | aUPD |  | UPD |  | Het del |  | Het del |  |  | Hom | Het del |
| 18 | T47D | Hom | aUPD | Het |  | Met | Het del |  |  |  | UPD |  | UPD |
| 19 | UACC812 | NM | Het Del |  |  | Met |  |  |  |  | UPD |  |  |
| 20 | ZR751 | NM | Het Del |  |  | Met |  | Hom | UPD |  | UPD |  | UPD |
| 21 | CAL120 | Hom | aUPD |  |  |  | Het del |  | Het del |  | Het del |  | UPD |
| 22 | CAL51 | NM |  | Het |  |  |  |  |  |  |  |  |  |
| 23 | CAL851 | Hom | aUPD |  |  |  |  |  | UPD | Hom | UPD |  |  |
| 24 | EFM19 | Het | aUPD | Hom | UPD | Hom | Het del |  | Het del |  |  |  | Het del |
| 25 | EFM192A | Hom | aUPD |  |  |  | Het del |  |  |  |  |  |  |
| 26 | EVSA-T | Hom | aUPD |  |  |  |  | Hom | Het del |  |  | Hom | UPD |
| 27 | HCC1937 | Hom | aUPD |  |  |  | UPD |  | Het del |  | Het del |  |  |
| 28 | KPL1 | NM |  |  |  |  | UPD |  |  |  |  |  | UPD |
| 29 | HCC202 | Hom | Het Del |  |  |  | UPD |  |  |  |  |  |  |
| 30 | HCC1187 | NA | aUPD |  | Het del |  | UPD |  | Het del |  | UPD |  | Het del |
| 31 | HCC1419 | Hom | aUPD |  |  |  | Het del |  |  |  |  |  |  |
| 32 | HCC70 | Hom | Het Del |  | UPD |  |  | Hom | UPD |  | UPD |  | UPD |
| 33 | HCC1428 | NM | Het Del |  |  |  | Het del |  |  |  | Het del |  |  |
| 34 | HCC1954 | Hom | aUPD | Het | UPD |  |  |  | Het del |  |  |  |  |
| 35 | HCC2218 | Hom | Het Del |  |  |  |  |  |  |  | Het del | Hom | Het del |
| 36 | HCC1500 | NM | Del |  | UPD |  | Hom del |  | Het del |  | Het del |  |  |
| 37 | HCC1395 | Hom | aUPD |  |  | Hom | UPD | Hom | Het del |  | Het del |  |  |
| 38 | HCC1143 | NM | aUPD |  |  |  | Het del |  |  |  | Het del |  |  |
| 39 | ZR7530 | NM | Het Del |  |  |  |  |  |  |  |  | Hom | Het del |
| 40 | MFM223 | Hom | aUPD | Het |  |  | Het del |  | UPD |  |  |  | UPD |
| 41 | HCC1569 | Het |  |  |  |  |  |  | UPD |  |  |  |  |
| 42 | HCC38 | Hom | aUPD |  | UPD | Hom | UPD |  | UPD |  |  |  |  |
| 43 | CAL148 | Het |  | Het |  |  |  |  |  | Hom |  |  | Het del |
| 44 | UACC893 | Hom | Het Del | Het |  | Met | Het del |  | Het del |  | Het del |  |  |

aUPD; acquired Uniparental Disomy, Hom; homozygous mutation, Het del; heterozygous deletion, Hom del; homozygous deletion, NM; no mutation, NA; not available, Met; promoter methylation. There are some discrepancy between SNP microarray and mutation data, for example HCC1954 cell lines harbor heterozygous mutation at *PIK3CA* and aUPD at same region (3q26.32), MDAMB231 cell lines harbor homozygous mutation and deletion at *CDKN2A* region, and DU4475 cell lines harbor homozygous mutation and deletion at *RB1* region, this may due to (1) different passage of cell lines used for sequencing and SNP microarray experiments since microarray and sequencing performed with different researchers and cells may change after passages, and (2) tumor heterogeneity. This data suggesting that, aUPD region may pinpoint not only homozygous mutation, but also homozygous methylation or histon modification.
