# Supplementary material for: Association between Acquired Uniparental Disomy and Homozygous Mutations and HER2/ER/PR Status in Breast Cancer
Source: PLoS One. 2010 Nov 30;5(11):e15094. doi: 10.1371/journal.pone.0015094 (PMC2994899; doi:10.1371/journal.pone.0015094)
Supplement: Table S6 — Previously reported homozygous mutated genes in breast cancer samples. (DOC) [file pone.0015094.s006.doc]

**Table S6.** Previously reported homozygous mutated genes in breast cancer samples.

| **Gene** | **RefSeq Accession*** | **Gene name** | **Function** | **Chromosome band** | **References for mutations** |
| --- | --- | --- | --- | --- | --- |
| *ABCA12* | NM_173076 | ATP-binding cassette, sub-family A, member 12 | Tansport various molecules | 2q35 | [1] |
| *ABP1* | NM_001091 | Ailoride binding protein 1 precursor | Cntrolling the level of histamine and/or putrescine | 7q36.1 | [1] |
| *AEGP* | NM_206920 | MAM domain containing 4 | Transport of receptors and ligands | 9q34.3 | [1,2] |
| *AGC1* | NM_001135 | Aggrecan | Etracellular matrix | 15q26.1 | [1] |
| *AGRN* | NM_198576 | Agrin | Agregating factor | 1p36.33 | [1] |
| *AKT1* | NM_005163 | Protein kinase B | Antiapoptotic | 14q32.33 |  |
| *AMPD2* | NM_139156.1 | Aenosine monophosphate deaminase 2 | Role in the purine nucleotide cycle | 1p13.13 | [1] |
| *APBB1* | NM_145689 | Amyloid beta A4 precursor protein-binding | Cell cycle, thymidylate synthase, nuclear translocation | 11p15.4 | [1] |
| *APXL* | NM_001649.2 | shroom family member 2 (SHROOM2), | Sodium channel activity | Xp22.2 | [1] |
| *ARHGEF4* | NM_015320 | Rho guanine nucleotide exchange factor (GEF) 4 | Cell migration, E-cadherin-mediated cell-cell adhesion | 2q21.1 | [1,2] |
| *ASGR1* | NM_001671.2 | Asaloglycoprotein receptor 1 | Transports glycoproteins | 17p13.1 | [1] |
| *ATCAY* | NM_033064 | Caytaxin |  | 19p13.3 | [1] |
| *ATP8B4* | NM_024837 | ATPase class I type 8B member 4 | Phospholipid-translocating ATPase activity | 15q21.2 | [1] |
| *ATR* | NM_001184 | Ataxia telangiectasia and Rad3 related (ATR), | Serine/threonine protein kinase, DNA replication, mitosis, DNA repair, recombination and apoptosis | 3q23 | [1,3] |
| *AVPI1* | NM_021732.1 | Vasopressin-induced protein |  | 10q24.2 | [1] |
| *BAP1* | NM_004656.2 | BRCA1 associated protein-1 | Tumor suppressor, growth control | 3p21.1 | [1] |
| *BAZ1A* | NM_013448.2 | BAZ1A protein | Chromatin remodeling, transcriptional regulation | 14q13.2 | [1] |
| *BC002942* | NM_033200.1 | Lipase maturation factor 2 | Maturation of specific proteins | 22q13.33 | [1] |
| *BMP1* | NM_006129.2 | Bone morphogenetic protein 1 | Cartilage and bone formation | 8p21.3 | [1] |
| *BRCA1* | NM_007297 | Breast cancer 1, early onset | Tumor suppressor, DNA damage repair, ubiquitination, genomic stability, cell cycle | 17q21.31 | [1,2] |
| *C10orf45* | NM_031453.2 | chromosome 10 open reading frame 45 | Hypothetical protein | 10p13 | [1] |
| *C14orf161* | NM_024764 | Cation channel sperm-associated protein | Protein binding, cell differentiation | 14q32.12 | [1] |
| *C14orf29* | NM_181814.1 | Abhydrolase domain containing 12B |  | 14q22.1 | [1] |
| *C14orf46* | NM_001024674 | lin-52 homolog (C. elegans) |  | 14q24.3 | [1] |
| *C1QB* | NM_000491.2 | Complement component 1, q subcomponent, B chain | Protein homodimerization activity | 1p36.12 | [1] |
| *C4orf14* | NM_032313.2 | Chromosome 4 open reading frame 14 | Hypothetical protein LOC84273 | 4q12 | [1] |
| *C6orf213* | NM_001010852 | Clavesin 2 | Lipid binding, transporter activity | 6q22.31 | [1] |
| *CCDC66* | NM_001012506 | Coiled-coil domain-containing protein 66 |  | 3p14.3 | [1] |
| *CDC27* | NM_001256.2 | Cell division cycle protein 27 | Cell cycle, ubiquitination | 17q21.32 | [1] |
| *CDH1* | NM_004360 | Epithelial cadherin | Cell-cell adhesion | 16q22.1 | COSMIC# |
| *CDKN2A* | NM_058197 | Cyclin-dependent kinase inhibitor 2A | cell cycle, apoptosis | 9p21.3 | COSMIC# |
| *CENTD3* | NM_022481.4 | ArfGAP with RhoGAP domain, ankyrin repeat and PH | Negative regulation of cell migration, signal transduction, cell shape | 5q31.3 | [1] |
| *CGI-37* | NM_016101.2 | Nuclear import 7 homolog (S. cerevisiae) | Pre-rrna processing | 16q22 | [1] |
| *CNGA2* | NM_005140.1 | Cyclic nucleotide gated channel alpha 2 | Nucleotide binding, ion transport | Xq28 | [1] |
| *CNTN6* | NM_014461 | Contactin 6 | Mediate cell surface interactions | 3p26.3 | [1,2] |
| *COG3* | NM_031431.2 | Component of golgi transport complex 3 | Er-golgi transport | 13q14.12 | [1] |
| *CROCC* | NM_014675 | Ciliary rootlet coiled-coil, rootletin | Centrosome cohesion | 1p36.13 | [1] |
| *CRSP2* | NM_004229.2 | Mediator complex subunit 14 | Component of the mediator complex | Xp11.4 | [1] |
| *CSMD1* | NM_033225 | CUB and Sushi multiple domains 1 | Potential suppressor of squamous cell carcinomas | 8p23.2 | [1] |
| *CTNNA1* | NM_001903 | Catenin, alpha 1 | Cell differentiation | 5q31.2 | [1] |
| *DDX10* | NM_004398 | DEAD (Asp-Glu-Ala-Asp) box polypeptide 10 | Putative ATP-dependent RNA helicase | 11q22.3 | [2] |
| *DDX3X* | NM_024005.1 | DEAD/H (Asp-Glu-Ala-Asp/His) box polypeptide 3 | RNA, DNA and protein binding, helicase and hydrolase activity | Xp11.4 | [1] |
| *DGKE* | NM_003647.1 | Diacylglycerol kinase epsilon | Diacylglycerol kinase activity | 17q22 | [1] |
| *DHX32* | NM_018180.2 | DEAD/H (Asp-Glu-Ala-Asp/His) box polypeptide 32 | ATP binding, helicase activity | 10q26.2 | [1] |
| *DMD* | NM_004006.1 | Dystrophin | Anchoring the cytoskeleton to the plasma membrane | Xp21.1 | [1] |
| *DNASE1L3* | NM_004944 | Deoxyribonuclease I-like 3 | DNA hydrolytic activity | 3p14.3 | [1,2] |
| *DTX3L* | NM_138287.2 | E3 ubiquitin-protein ligase DTX3L | Ubiquitin ligase | 3q21.1 | [1] |
| *DUOX1* | NM_017434 | Dual oxidase 1 precursor | Thyroid hormones synthesis | 15q21.1 | [1] |
| *DYSF* | NM_003494.2 | Dysferlin | Membrane regeneration and repair | 2p13.3 | [1] |
| *FANCM* | NM_020937 | Fanconi anemia, complementation group M | DNA repair | 14q21.3 | [1] |
| *FARP1* | NM_005766.1 | FERM, RhoGEF, and pleckstrin domain protein 1 | Rho-guanine nucleotide exchange factor | 13q32.2 | [1] |
| *FGFR1* | NM_023106 | Fibroblast growth factor receptor 1 | Fms-like tyrosine kinase 2 | 8p12 | [1,3] |
| *FLJ10241* | NM_018035 | ATP5S-like | ATP synthase | 19q13.2 | [1] |
| *FLJ13089* | NM_024953.2 | Nap1 | Protein binding | 12q24.13 | [1] |
| *FLJ21839* | NM_021831.3 | ATP/GTP binding protein-like 5 | Proteolysis | 2p23.3 | [1] |
| *FLJ32830* | NM_152781.1 | Hypothetical protein |  | 17q12 | [1] |
| *FLJ34521* | NM_001039787 | Hypothetical protein |  | 11q24.3 | [1] |
| *FLJ46481* | NM_207405.1 | Hypothetical protein |  | 4p16.1 | [1] |
| *FLNA* | NM_001456 | Filamin A | Intracellular trafficking | Xq28 | [1] |
| *FMNL3* | NM_175736 | Formin-like 3 | Protein binding, cellular component organization | 12q13.12 | [1] |
| *GALK2* | NM_001001556 | Galactokinase 2 | Kinase activity | 15q21 | [1] |
| *GGA3* | NM_014001.2 | ADP-ribosylation factor binding protein 3 | Intracellular protein transport | 17q25.1 | [1] |
| *GMCL1L* | NM_022471.2 | Germ cell-less homolog 1 (Drosophila)-like (GMCL1L), | Nucleocytoplasmic transport (enhances the degradation of MDM2 and increases the amount of p53) | 5q35.3 | [1] |
| *GNB1L* | NM_053004.1 | Guanine nucleotide binding protein (G protein), beta polypeptide 1-like (GNB1L) | Intracellular signaling cascade | 22q11.21 | [1] |
| *GOLGB1* | NM_004487.1 | Golgin B1 (GOLGB1), | Golgi organization | 3q13.33 | [1] |
| *GPC1* | NM_002081.1 | Glypican 1 precursor | Heparan sulfate proteoglycan binding | 2q37.3 | [1] |
| *GPC2* | NM_152742.1 | Glypican 2 | Heparan sulfate proteoglycan binding | 7q22.1 | [1] |
| *GRIPAP1* | NM_207672 | GRIP1 associated protein 1 | Not known | Xp11.23 | [1] |
| *GSDML* | NM_018530.1 | Gasdermin-like | Apoptosis | 17q12 | [1] |
| *GUCY2F* | NM_001522 | Guanylate cyclase 2F | Photoreceptors in the retina | Xq22.3-q23 | [1,3] |
| *HK3* | NM_002115.1 | Hexokinase 3 | Kinase activity | 5q35.2 | [1] |
| *HOOK2* | NM_013312 | Hook homolog 2 | Protein transport | 19p13.3 | [1] |
| *ICAM5* | NM_003259 | Intercellular adhesion molecule 5, telencephalin | Adhesion | 19p13.2 | [1,2] |
| *IQSEC2* | NM_015075 | IQ motif and Sec7 domain 2 | ARF guanyl-nucleotide exchange factor activity | Xp11.22 | [1] |
| *ITGA9* | NM_002207.1 | Integrin, alpha 9 precursor | Cell adhesion, integrin-mediated signaling pathway | 3p22.2 | [1] |
| *JMJD1C* | NM_004241 | Jumonji domain containing 1C | Chromatin modification, regulation of transcription | 10q21.2 | [1] |
| *KCNJ1* | NM_000220.2 | Potassium inwardly-rectifying channel J1 | Nucleotide binding, potassium ion transport | 11q24.3 | [1] |
| *KIAA0863* | NM_014913 |  |  | 18q23 | [1] |
| *KIAA0913* | NM_015037 | Hypothetical protein | Zinc ion binding | 10q22.2 | [1] |
| *KIAA1377* | NM_020802 | Hypothetical protein | Protein binding | 11q22.1 | [1] |
| *KIAA1797* | NM_017794 | Hypothetical protein | Receptor binding | 9p21.3 | [1] |
| *LOC200420* | NM_145300 |  |  | 2p13.2 | [1] |
| *LOC388915* | NM_001010902 |  |  | 22p21 | [1] |
| *LOC440925* | NM_001013712 | Hypothetical protein |  | 2q31.1 | [1] |
| *LPO* | NM_006151 | Lactoperoxidase | Response to oxidative stress | 17q22 | [1] |
| *LRBA* | NM_006726 | LPS-responsive vesicle trafficking, beach and anchor containing | Vesicle trafficking | 4q31.3 | [1,2] |
| *LRRC7* | NM_020794.1 | Leucine rich repeat containing 7 | Focal adhesion | 1p31.1 | [1] |
| *LRRFIP1* | NM_001137550 | Transcription factor 9-like | Transcriptional repressor, cells proliferation | 2q37.3 | [1] |
| *MAGEB10* | NM_182506 | Melanoma antigen family B, 10 | Not known | Xp21.3 | [1] |
| *MAGEE1* | NM_020932 | Hepatocellular carcinoma-associated protein 1 | Not known | Xq13.3 | [1,2] |
| *MAOA* | NM_000240.2 | Monoamine oxidase A | Oxidoreductase activity, protein binding | Xp11.3 | [1] |
| *MAPKBP1* | NM_014994 | Mitogen-activated protein kinase-binding protein 1 | Protein kinase | 15q15.1 | [1] |
| *MCART1* | NM_033412.1 | Mitochondrial carrier triple repeat 1 | Not known | 9p13.2 | [1] |
| *MED12* | NM_005120 | Mediator complex subunit 12 | Regulation of transcription | Xq13.1 | [1] |
| *MET* | NM_000245 | Met proto-oncogene (hepatocyte growth factor receptor) | Cell proliferation, scattering, morphogenesis and survival | 7q31.2 | [1,3] |
| *MGC24047* | NM_178840 | Chromosome 1 open reading frame 64 | Not known | 1p36.13 | [1,2] |
| *MIA2* | NM_054024.3 | Melanoma inhibitory activity 2 | Not known | 14q21.1 | [1] |
| *MPFL* | NM_001025190 | Mesothelin-like (MSLNL), | Cell adhesion | 16p13.3 | [1] |
| *MTA1* | NM_004689 | Metastasis associated protein | Transcription factor | 14q32.33 | [1] |
| *MUC2* | NM_002457 | Mucin 2 precursor | Protein binding | 11p15.5 | [1] |
| *MYH1* | NM_005963 | Myosin, heavy polypeptide 1, skeletal muscle, adult | Muscle contraction | 17p13.1 | [1,2] |
| *MYO3A* | NM_017433 | Myosin IIIA | Vision and hearing | 10p12.1 | [1,3] |
| *MYO15A* | NM_016239 | Myosin XV | Protein binding | 17p11.2 | [1] |
| *MYO1G* | NM_033054 | Myosin IG | Nucleotide binding | 7p13 | [1] |
| *MYST4* | NM_012330.1 | MYST histone acetyltransferase (monocytic leukemia) 4 | Histone acetyltransferase activity, regulation of transcription | 10q22 | [1] |
| *NALP1* | NM_014922 | NLR family, pyrin domain containing 1 | Apoptosis | 17p13.2 | [1] |
| *NDUFA2* | NM_002488.2 | NADH dehydrogenase (ubiquinone) 1 alpha | Electron transfer | 5q31.3 | [1] |
| *NFKBIA* | NM_020529.1 | Nuclear factor of kappa light polypeptide gene | Transcription factor binding | 14q13.2 | [1] |
| *NHS* | NM_198270.2 | Nance-Horan syndrome protein | Not known | Xp22.1 | [1] |
| *NUFIP2* | NM_020772 | Nuclear fragile X mental retardation protein | Protein binding | 17q11.2 | [1] |
| *NUP133* | NM_018230 | Nucleoporin 133kDa | Poly(A)+ RNA transport | 1q42.13 | [1,2] |
| *NUP98* | NM_016320.2 | Nucleoporin 98kD | Transporter activity | 11p15.4 | [1] |
| *OR4D2* | NM_001004707 | Olfactory receptor, family 4, subfamily D | Receptor activity | 17q22 | [1] |
| *OR52H1* | NM_001005289 | Olfactory receptor, family 52, subfamily H | Receptor activity | 11p15.4 | [1] |
| *OR5H1* | NM_001005338 | Olfactory receptor, family 5, subfamily H, member 1 | Receptor activity | 3q11.2 | [1] |
| *OR8D2* | NM_001002918 | Olfactory receptor, family 8, subfamily D, member 2 | Receptor activity | 11q24.2 | [1] |
| *OTOF* | NM_194232 | Otoferlin | Calcium ion sensor | 2p23.3 | [1,2] |
| *PADI3* | NM_016233.1 | Peptidylarginine deiminase type III | Protein-arginine deaminase activity | 1p36.13 | [1] |
| *PANX2* | NM_052839.2 | Pannexin-2 | Protein binding | 22q13.33 | [1] |
| *PAPPA2* | NM_020318 | Pappalysin 2 | Growth, dfferentiation | 1q25.2 | [1] |
| *PCDH19* | NM_020766 | Protocadherin 19 | Adhesion | Xq22.1 | [1] |
| *PCDH8* | NM_002590.2 | Protocadherin 8 | Adhesion, cell-cell signaling | 13q21.1 | [1] |
| *PCDHB15* | NM_018935 | Protocadherin beta 15 | Adhesion | 5q31.3 | [1,2] |
| *PCM1* | NM_006197 | Pericentriolar material 1 | Centrosome organization | 8p22 | [1] |
| *PDCD11* | NM_014976 | Programmed cell death 11 | RNA processing, transcription factor binding | 10q24.33 | [1] |
| *PER1* | NM_002616.1 | Period homolog 1 (Drosophila) (PER1), | Transcription regulation | 17p13.1 | [1] |
| *PEX14* | NM_004565 | Peroxisomal biogenesis factor 14 | Transcription corepressor activity | 1p36.22 | [1] |
| *PFKFB4* | NM_004567.2 | 6-phosphofructo-2-kinase/fructose-2, 6-biphosphatase 4 | Kinase activity | 3p21.31 | [1] |
| *PIK3C2G* | NM_004570 | Phosphoinositide-3-kinase, class 2, gamma | Kinase activity, proliferation, oncogenic transformation, survival, migration | 12p12.3 | [1] |
| *PIK3CA* | NM_006218 | Phosphatidylinositol 3-kinase, catalytic, 110-KD, alpha | Inositol or phosphatidylinositol kinase activity | 3q26.32 | [1],  COSMIC # |
| *PKD1L2* | NM_052892 | Polycystic kidney disease protein 1-like 2 precursor (Polycystin-1L2) | Ion transport | 16q23.2 | [1] |
| *PKDREJ* | NM_006071 | Polycystic kidney disease (polycystin) and REJ (sperm receptor for egg jelly homolog, sea urchin)-like | Ca(2+) transporting | 22q13.31 | [2] |
| *PPHLN1* | NM_201439.1 | Periphilin 1 | Differentiation | 12q12 | [1] |
| *PPP1R12A* | NM_002480 | Protein phosphatase 1, regulatory (inhibitor) subunit 12A | Signal transducer activity | 12q21.31 | [1] |
| *PRDM4* | NM_012406.3 | PR domain containing 4 | Proliferaion, growth, cell cycle, regulation of transcription | 12q23.3 | [1] |
| *PTCH1* | NM_000264 | Patched homolog 1 (Drosophila) | receptor for sonic hedgehog, tumor suppressor | 9q22.32 |  |
| *PTD004* | NM_013341.2 | GTP-binding protein PTD004 | Hydrolase activity, protein binding | 2q31.1 | [1] |
| *PTEN* | NM_000314 | Phosphatase and tensin homolog | Tumor suppressor, protein phosphatase, dephosphorylating  tyrosine-, serine- and threonine-phosphorylated proteins, lipid phosphatase, cell cycle, survival, migration, adhesion | 10q23.31 | [1], COSMIC# |
| *PURG* | NM_013357.2 | Purine-rich element binding protein G | DNA replication and transcription | 8p12 | [1] |
| *PUS1* | NM_025215.3 | Pseudouridylate synthase 1 | RNA binding, pseudouridine synthase activity | 12q24.33 | [1] |
| *RANBP3* | NM_007322 | RAN binding protein 3 | Protein transport | 19p13.3 | [1] |
| *RASL10B* | NM_033315.2 | RAS-like, family 10, member B | GTPase activity | 17q12 | [1] |
| *RBAF600* | NM_020765.1 | Retinoblastoma-associated factor 600 | Ubiquitin-protein ligase activity | 1p36.13 | [1] |
| *RFX2* | NM_000635 | Regulatory factor X, 2 | Transcription factor | 19p13.3 | [1,2] |
| *RLTPR* | NM_001013838 | RGD motif, leucine rich repeats, tropomodulin domain and proline-rich containing | Not known | 16q22.1 | [1] |
| *RNU3IP2* | NM_004704 | RNA, U3 small nucleolar interacting protein 2 | Processing and modification of pre-ribosomal RNA | 3p21.2 | [1,2] |
| *RP1L1* | NM_178857 | Retinitis pigmentosa 1-like 1 | Intracellular signaling cascade | 8p23.1 | [1] |
| *RPRC1* | NM_018067 | MAP7 domain containing 1 | Cell division, endocytosis | 1p34.3 | [1] |
| *RTP1* | NM_153708.1 | Receptor transporting protein 1 | Olfactory receptor binding | 3q27.3 | [1] |
| *SCGB3A2* | NM_054023.2 | Secretoglobin, family 3A, member 2 | Protein binding | 5q33.1 | [1] |
| *SEMA5B* | NM_018987.1 | Semaphorin 5B | Differentiation | 3q21.1 | [1] |
| *SH2D3A* | NM_005490.1 | SH2 domain containing 3A | Guanyl-nucleotide exchange factor activity | 19p13.3 | [1] |
| *SH3RF1* | NM_020870 | SH3 domain containing ring finger 1 | Apoptosis, ligase activity | 4q32.3-q33 | [1] |
| *SIX4* | NM_017420 | Sine oculis homeobox homolog 4 | Not known | 14q23.1 | [2] |
| *SLC17A6* | NM_020346.1 | Solute carrier family 17 (sodium-dependent inorganic phosphate cotransporter), member 6 | Sodium ion transport, cell junction, Cytoplasmic vesicle | 11p14.3 | [1] |
| *SLC8A3* | NM_182932.1 | Solute carrier family 8 (sodium/calcium exchanger), member 3 | Calcium ion transport, cell communication | 14q24.2 | [1] |
| *SNTG2* | NM_018968 | Syntrophin, gamma 2 | Protein binding | 2p25.3 | [1] |
| SORL1 | NM_003105 | Sortilin-related receptor, L (DLR class) A repeats-containing | Cell-cell interaction | 11q23.3 | [2] |
| SPATS2 | NM_023071 | Spermatogenesis associated, serine-rich 2 | Not known | 12q13.12 | [1] |
| STAB1 | NM_015136 | Stabilin 1 precursor | Adhesion, cell-cell interaction | 3p21.1 | [1] |
| *STARD8* | NM_001142503 | START domain containing 8 | GTPase activity | Xq13.1 | [1,2] |
| *SYNE2* | NM_015180 | Spectrin repeat containing, nuclear envelope 2 | Nuclear organization | 14q23.2 | [1,2] |
| *TAF1* | NM_138923 | Transcription initiation factor TFIID 250 kDa subunit | Transcription initiation, cell cycle | Xq13.1 | [1,3] |
| *TCF1* | NM_000545 | Transcription factor 1, hepatic | Transcription factor | 12q24.31 | [1,2] |
| *TECTA* | NM_005422 | Tectorin alpha | Not known | 11q23.3 | [1,2] |
| *TESK1* | NM_006285 | Testis-specific protein kinase 1 | Serine/threonine protein kinase | 9p13.3 | [1,3] |
| *TG* | NM_003235 | Thyroglobulin | Signal transduction | 8q24.22 | [1] |
| *THBS3* | NM_007112 | Thrombospondin 3 | Cell-to-cell and cell-to-matrix interactions | 1q22 | [2] |
| *TIAM2* | NM_001010927 | T-cell lymphoma invasion and metastasis 2 | Apoptosis, Guanyl-nucleotide exchange factor activity, cell development | 6q25.2q25.3 | [1] |
| *TLN1* | NM_006289 | Talin 1 | Adhesion, migration, cell-cell junction | 9p13.3 | [1] |
| *TMEM62* | NM_024956 | Transmembrane protein 62 | Not known | 15q15.2 | [1] |
| *TMPRSS6* | NM_153609 | Transmembrane protease, serine 6 | Matrix remodeling | 22q12.3-q13.1 | [1,2] |
| *TP53* | NM_000546 | Tumor protein p53 | Apoptosis, cell cycle regulation | 17p13.1 | [1,2] |
| *TTN* | NM_133378 | Titin isoform N2-B | Chromosome condensation and segregation | 2q31.2 | [1] |
| *UBR4* | NM_020765 | Retinoblastoma-associated factor 600-like protein | Membrane morphogenesis, cytoskeletal organization, cell-matrix interactions | 1p36.13 |  |
| *UGT1A9* | NM_021027.2 | UDP glycosyltransferase 1 family, polypeptide A9 | Glucuronidase activity | 2q37.1 | [1] |
| *UQCR* | NM_006830.2 | Ubiquinol-cytochrome c reductase | Ubiquinol-cytochrome-c reductase activity, transport | 19p13.3 | [1] |
| *UTS2R* | NM_018949.1 | Urotensin 2 receptor | Cell growth, angiogenesis, proliferation | 17q25.3 | [1] |
| *WBP4* | NM_007187.3 | WW domain-containing binding protein 4 | RNA splicing | 13q14.11 | [1] |
| *WDR48* | NM_020839 | WD repeat domain 48 | Protein deubiquitination | 3p22.2 | [1] |
| *XDH* | NM_000379 | Xanthine dehydrogenase | Oxidative metabolism | 2p23.1 | [1,2] |
| *ZNF142* | NM_005081 | Zinc finger protein 142 | Transcription regulation | 2q35 | [1] |
| *ZNF183* | NM_006978.1 | Ring finger protein 113A | Protein binding, nucleic acid binding | Xq24 | [1] |

#Mutation was retrieved from **COSMIC** database (http://www.sanger.ac.uk/genetics/CGP/cosmic)

Gene accession * is Stanford Source.

1. Wood LD, Parsons DW, Jones S, Lin J, Sjoblom T, et al. (2007) The genomic landscapes of human breast and colorectal cancers. Science 318: 1108-1113.

2. Sjoblom T, Jones S, Wood LD, Parsons DW, Lin J, et al. (2006) The consensus coding sequences of human breast and colorectal cancers. Science 314: 268-274.

3. Stephens P, Edkins S, Davies H, Greenman C, Cox C, et al. (2005) A screen of the complete protein kinase gene family identifies diverse patterns of somatic mutations in human breast cancer. Nat Genet 37: 590-592.
