# Supplementary figures and images for: Association between Acquired Uniparental Disomy and Homozygous Mutations and HER2/ER/PR Status in Breast Cancer
Source: PLoS One. 2010 Nov 30;5(11):e15094. doi: 10.1371/journal.pone.0015094 (PMC2994899; doi:10.1371/journal.pone.0015094)

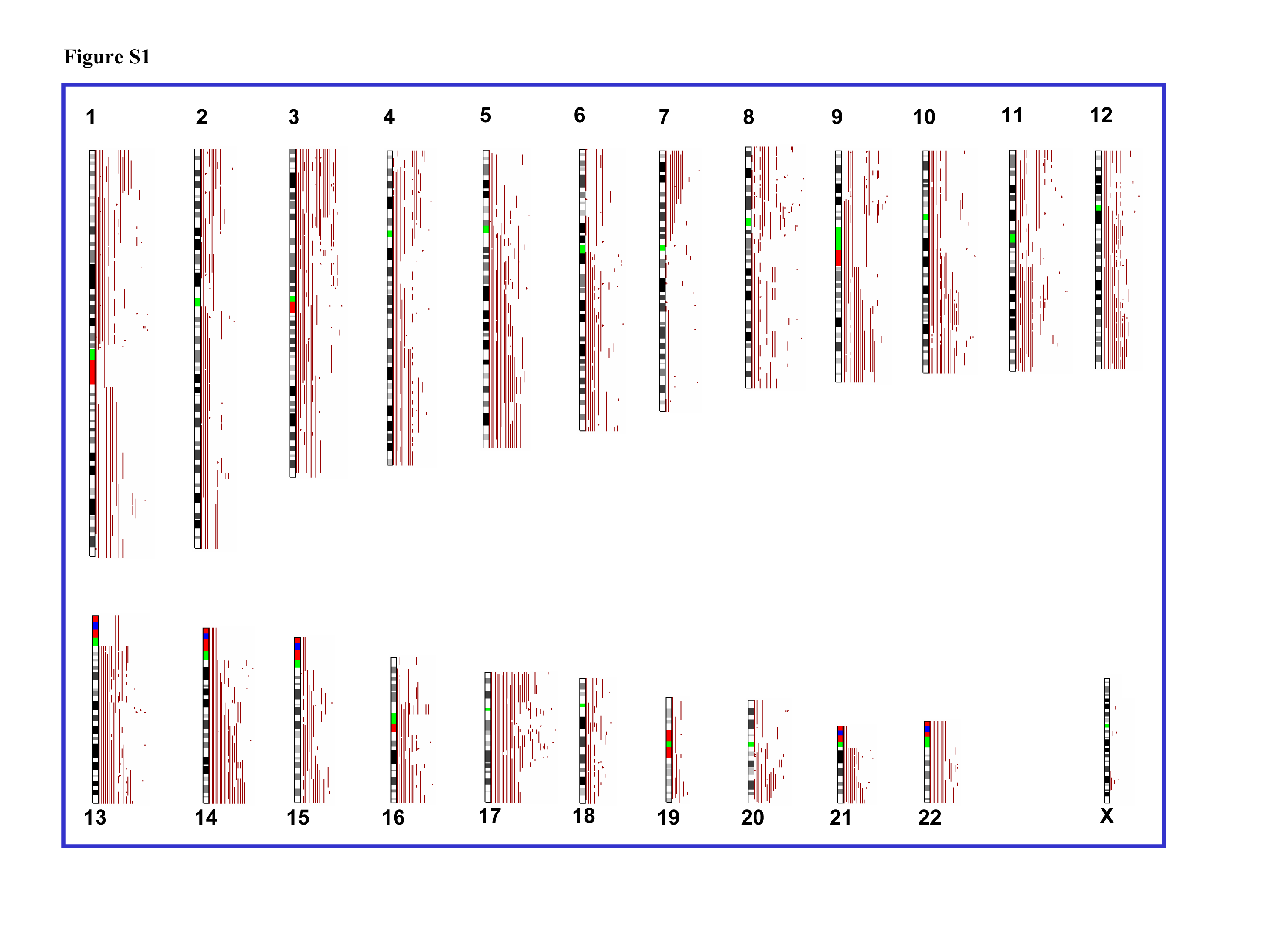

Supplement: Figure S1 — Distribution of aUPD regions in breast cancer cell lines (BrCaCL). (TIF) [file pone.0015094.s007.tif]

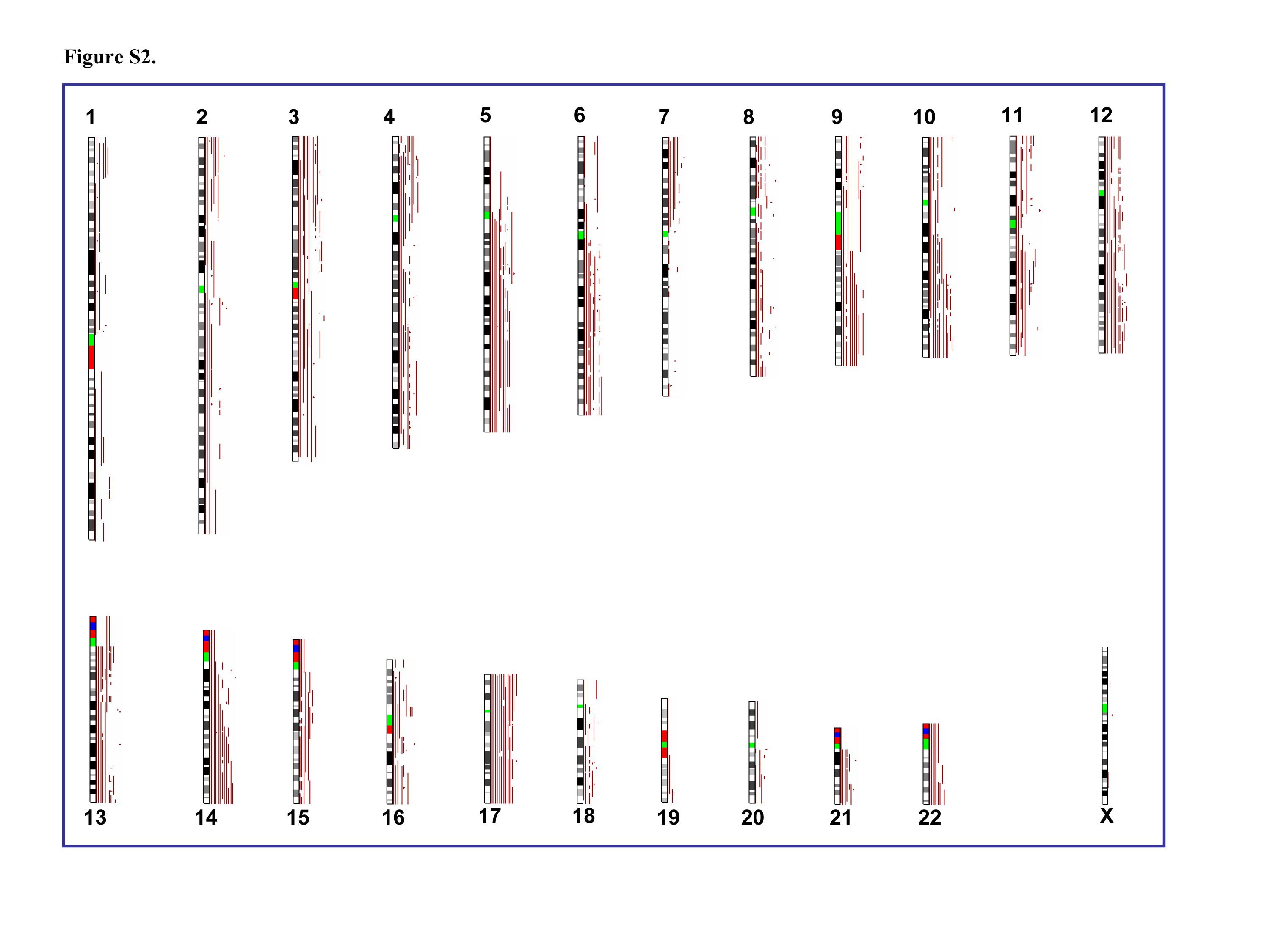

Supplement: Figure S2 — Distribution of aUPD regions in triple negative (BrCaCL). (TIF) [file pone.0015094.s008.tif]

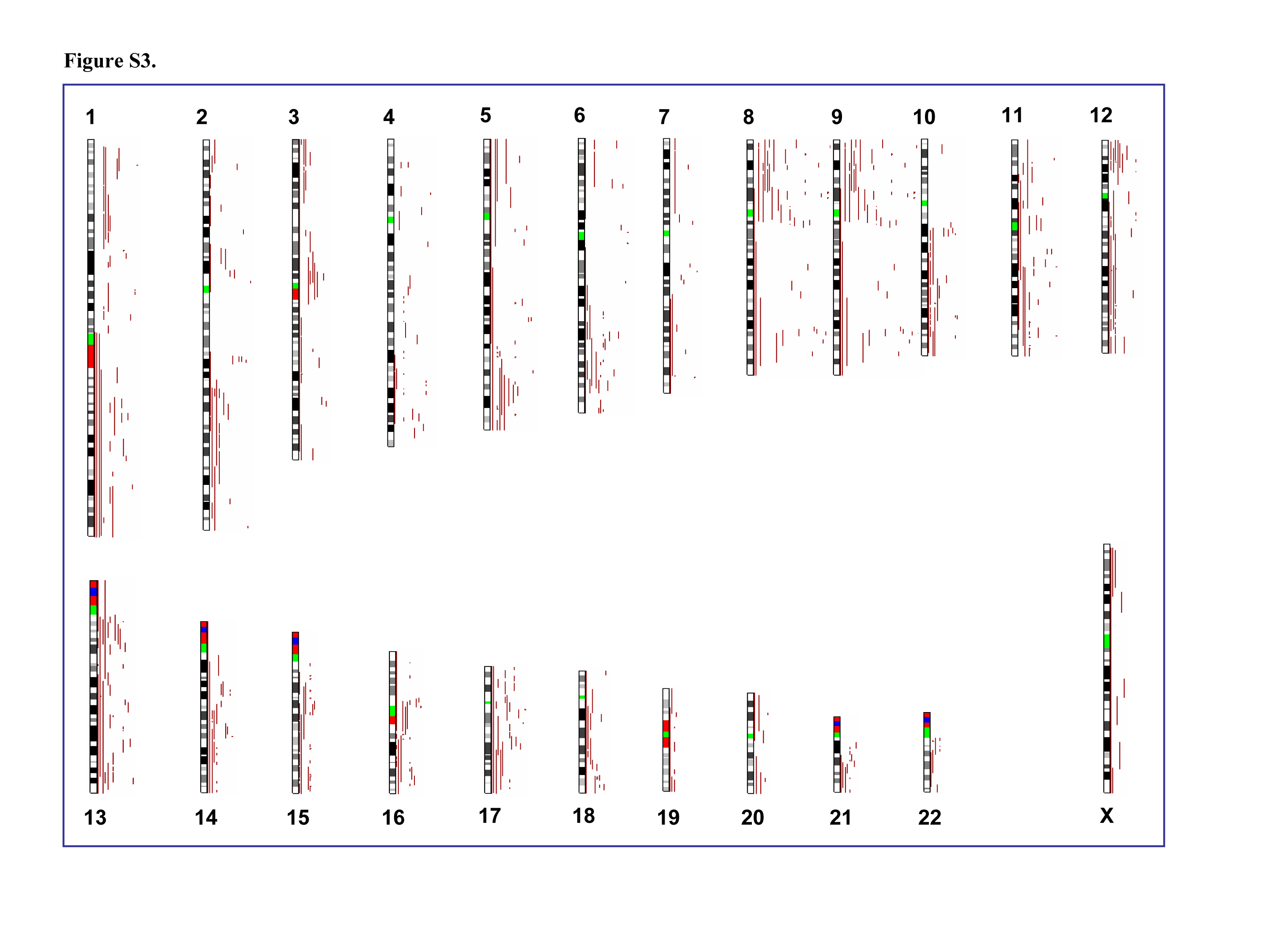

Supplement: Figure S3 — Distribution of aUPD in HER2/neu-positive BrCaCL. (TIF) [file pone.0015094.s009.tif]

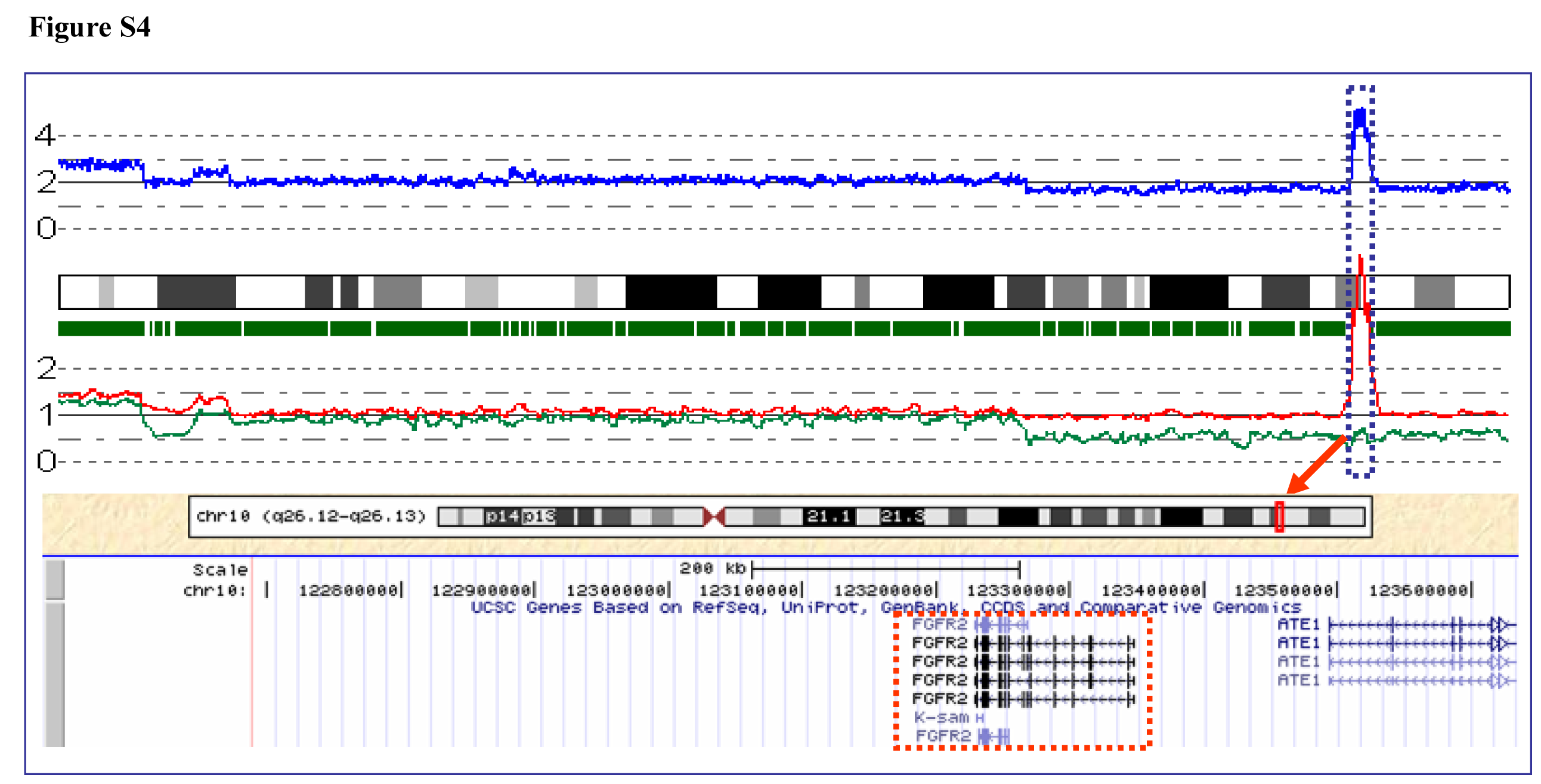

Supplement: Figure S4 — Representative smallest aUPD regions with focal amplification in TN samples. The upper panel represents total copy number (log2 ratio), on the middle chromosome idiogram, and green bar in the middle represents heterozygous SNP calls in tumor. The lower panel represents allele-based changes. At the bottom panel genes (FGFR2, ATE1 and K-sam) localized in the aUPD region at chromosome 10q26.12-q26.13. (TIF) [file pone.0015094.s010.tif]

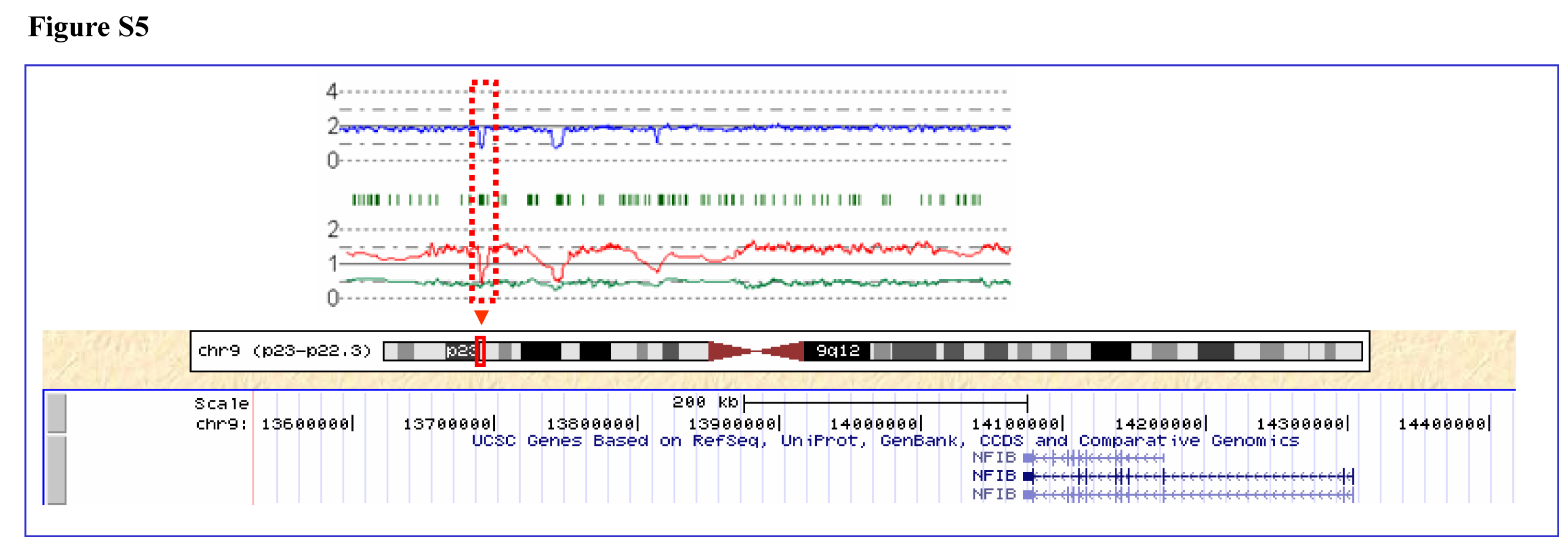

Supplement: Figure S5 — Representative smallest aUPD regions with homozygous deletion in TN samples. The upper panel represents total copy number (log2 ratio), on the middle chromosome idiogram, and green bar in the middle represents heterozygous SNP calls in tumor. The lower panel represents allele-based changes.gene (NFIB) at chromosome 9p23-p22.3 from genome browser (UCSC). (TIF) [file pone.0015094.s011.tif]

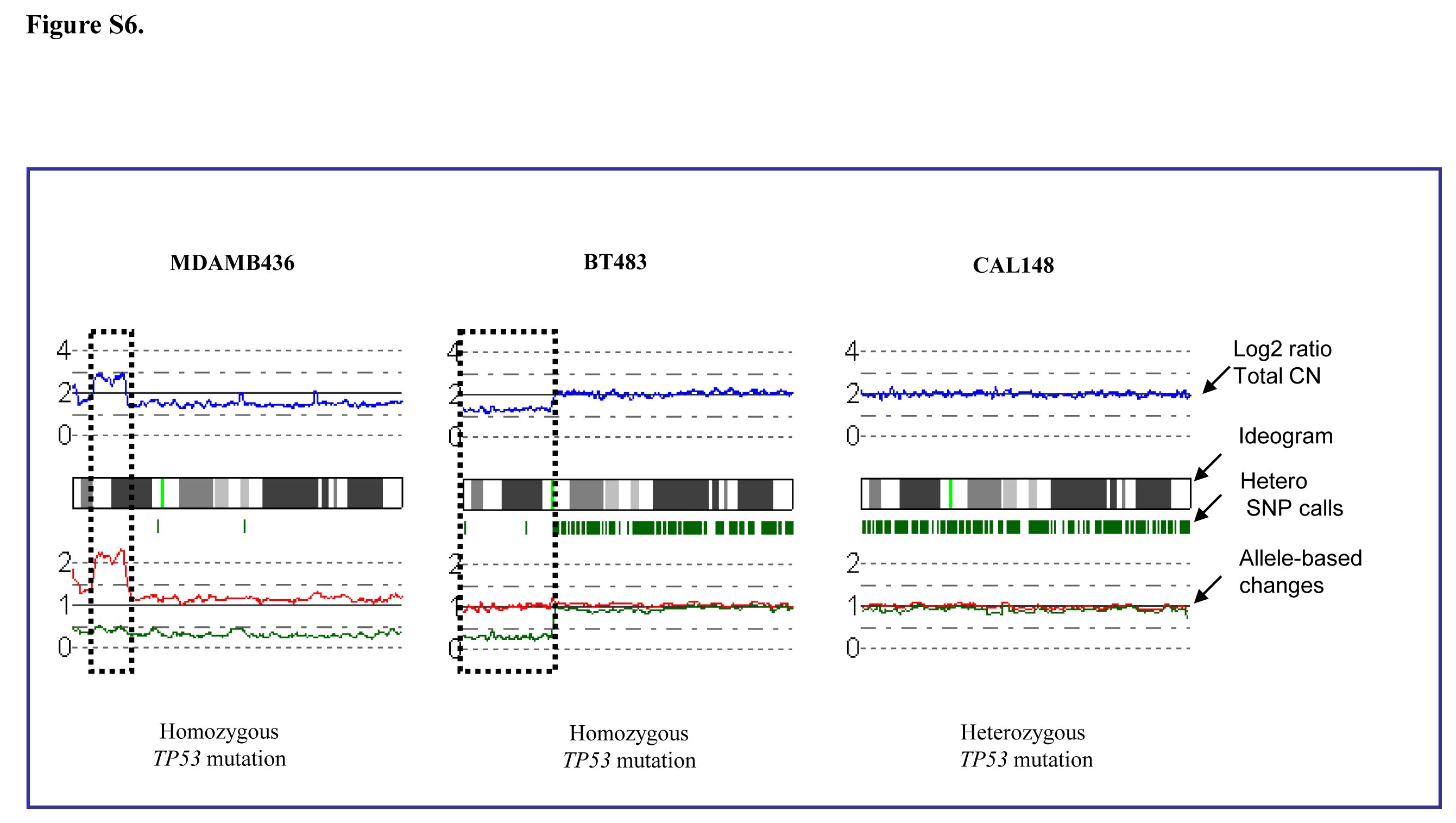

Supplement: Figure S6 — Representative figure for correlation of genomic and genetic data for three cell lines; MDAMB436, BT483 and CAL148. The upper panel showed log2 ratio, middle panel shoed chromosome ideogram and SNP heterozygous bar (green), and lower panel showed allele-based changes. Dashed square represents aUPD region at chromosome 17p. The first cell line harbors homozygous mutation for TP53 and aUPD at the same region. The second cell line harbors homozygous mutation for TP53 and heterozygous deletion at the same region. The third cell lines harbor heterozygous mutation for TP53 and no copy number changes. (TIF) [file pone.0015094.s012.tif]

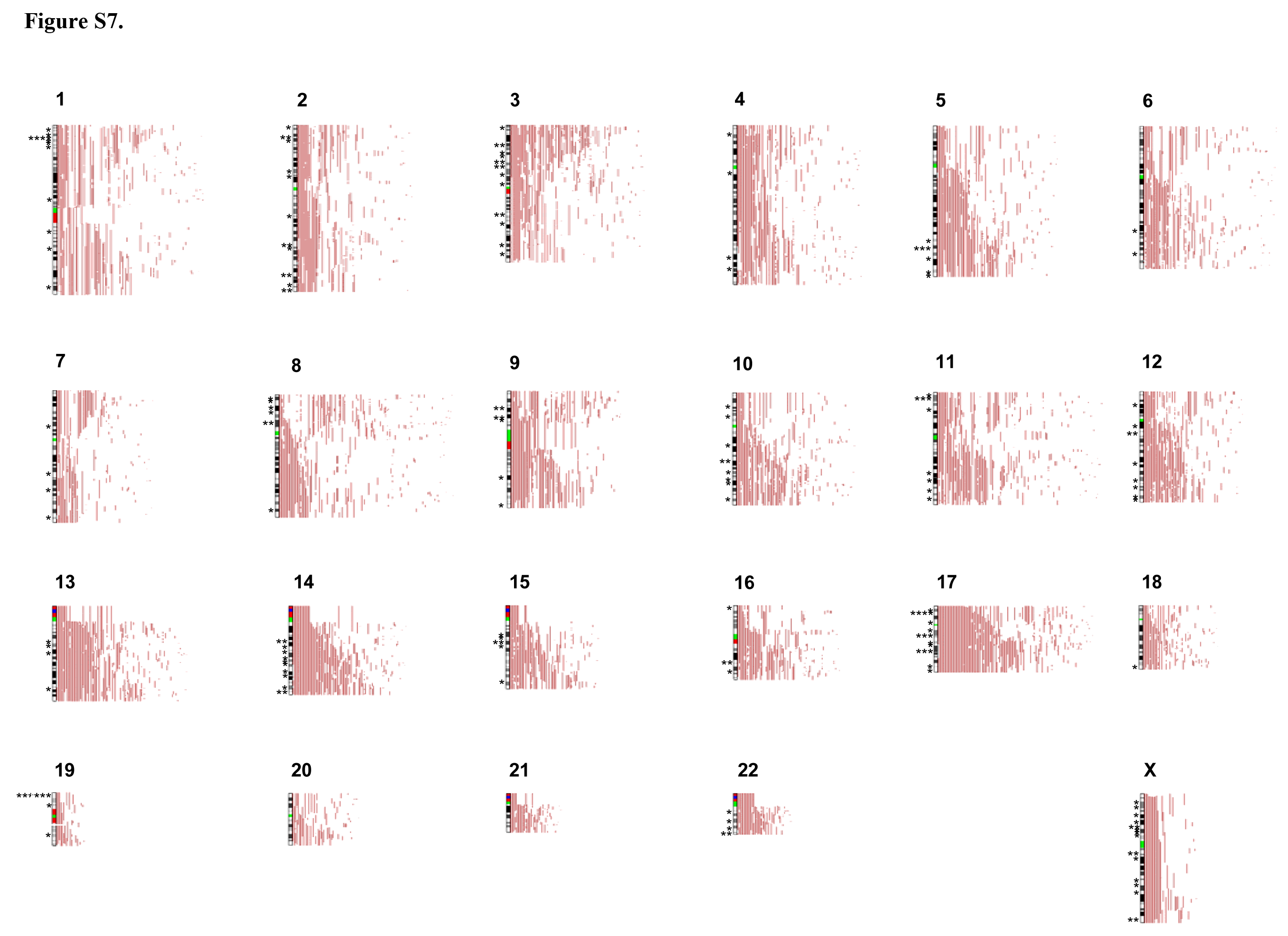

Supplement: Figure S7 — Distribution of aUPD and localization of previously reported homozygously mutated genes in breast cancer samples. Each line represents aUPD for each case. Each star represents previously reported homozygous mutated genes, which are also mapped in the aUPD regions in breast cancer. (TIF) [file pone.0015094.s013.tif]
